# Chronic Inflammatory Demyelinating Polyradiculoneuropathy During the COVID-19 Pandemic: Telemedicine Limitations and Strategies for Improvement

**DOI:** 10.7759/cureus.14146

**Published:** 2021-03-27

**Authors:** Adeel S Zubair, Aarij S Zubair, Kunal Desai

**Affiliations:** 1 Neurology, Yale School of Medicine, New Haven, USA; 2 Psychiatry, St. John's University, New York, USA

**Keywords:** cidp, covid-19, telemedicine (tm), chronic inflammatory demyelinating neuropathy

## Abstract

Chronic Inflammatory Demyelinating Polyneuropathy (CIDP) is a relapsing-remitting or progressive inflammatory neuropathy, which can present in a multitude of phenotypes. It can be a challenging condition to diagnose and requires thorough clinical evaluation and electrodiagnostic testing. With the outbreak of coronavirus disease in 2019 (COVID-19), large portions of the medical field converted to telemedicine to facilitate patient visits. We report a case of a 50-year-old female who was seen via video visit during the COVID-19 pandemic who was later diagnosed with CIDP and treated with intravenous immunoglobulins with improvement in clinical examination and electrodiagnostic testing. This case highlights the limitations of performing the neuromuscular examination via telemedicine.

## Introduction

Chronic Inflammatory Demyelinating Polyradiculoneuropathy (CIDP) is a relapsing-remitting or progressive inflammatory demyelinating polyneuropathy, which has a varied clinical presentation [1]. It has an estimated prevalence of 0.8 to 9 cases per 100,000 and is more common in men [2-4]. CIDP can be a challenging diagnosis for physicians due to the heterogeneity of presentations, ranging from distal vs proximal onset, symmetric vs asymmetric onset, and sensory vs motor variants. In 2010, the European Federation of Neurological Societies/Peripheral Nerve Society (EFNS/PNS) revised their 2006 criteria which they validated in multicenter European cohorts and have become the standards for clinical care [5]. The EFNS/PNS criteria define typical CIDP as “having proximal and distal weakness and sensory dysfunction of all extremities;” this is compared to atypical CIDP in which there can be predominantly distal asymmetric or focal symptoms, including pure motor or sensory [1, 5]. Other supportive criteria include elevation of protein in cerebrospinal fluid (CSF), response to treatment, and exclusion of other conditions [1, 5]. These criteria rely on clinical features and electrophysical evidence (conduction block, temporal dispersion) of demyelination to diagnose CIDP. Yet, even with these standards, misdiagnosis of CIDP is often seen.

The coronavirus disease 2019 (COVID-19) pandemic resulted in paradigm shifts in medical practice and clinical care. During the early stages of the pandemic, most non-acute care transitioned to virtual visits using technology such as video or telephone to conduct visits. This resulted in limited physical examination information which could be acquired by the provider.

This report discusses the case of a woman who presented via a virtual visit for symptoms of tingling in her extremities and was later diagnosed and treated for CIDP with clinical improvement.

## Case presentation

A 50-year-old female with a past medical history of anxiety was referred to the neurology clinic due to symptoms of paresthesias in her hands and feet. She reported that she had paresthesias in her fingers for the year prior to presentation which gradually progressed to symptoms in her feet over the course of the last few months. She described a sensation of burning in her feet as well. Prior to coming to the neurologist, she had tried acetaminophen, non-steroidal anti-inflammatory agents, gabapentin and duloxetine, which did not provide significant improvement. She had been supplementing with zinc, iron, calcium, biotin, and other vitamins.

Due to the COVID-19 pandemic, the patient participated virtually in the visit via secure tele-video services. Examination was limited due to the visit modality but included a normal mental status examination with intact orientation, concentration, and attention. The patient had fluent speech with no difficulties naming objects or repeating commands. Cranial nerve examination demonstrated intact extraocular muscle function, intact facial symmetry, and no dysarthria. Motor examination did not show any apparent muscle atrophy or obvious weakness or asymmetry, and the patient had a normal gait with normal based stance. Functional testing was attempted by having the patient go up and down a set of stairs. While she was able to perform this task, the visual observation was limited by the placement of the camera. Reflexes and sensation could not be assessed.

On the day of the visit, the patient had a severe acute respiratory syndrome coronavirus-2 (SARS-CoV-2) polymerase chain reaction (PCR) done due to an exposure which resulted positive, though the patient was asymptomatic. She was not started on any treatment for COVID-19. Other laboratory testing done prior to the visit included a normal C-reactive protein (CRP), erythrocyte sedimentation rate (ESR), vitamin B1, hemoglobin A1c, low-density lipoprotein, and thyroid-stimulating hormone. Vitamin B6 was elevated at 83 ng/mL (normal: 2.1-21.7 ng/mL). Based on the history and limited examination, it was felt that the patient had a sensory neuropathy of unclear etiology with the possibility of a superimposed median entrapment neuropathy (carpal tunnel syndrome). Due to her COVID diagnosis as well as patient preference, the plan was made to follow-up for a face-to-face visit in four weeks with an electromyography/nerve conduction study (EMG/NCS). Additionally, further laboratory workup including Lyme disease, antineutrophil cytoplasmic antibodies (ANCA), zinc, copper, hepatitis panel, and serum protein electrophoresis with immunofixation were ordered, all of which resulted normal.

**Figure 1:**
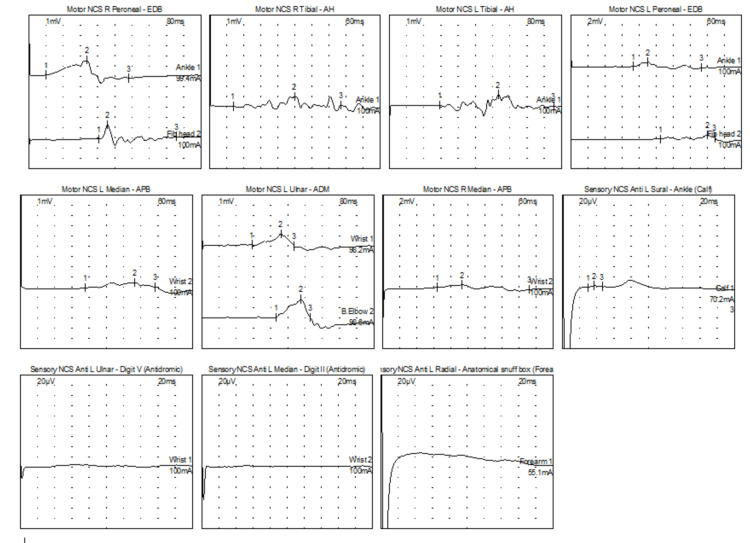
Initial electromyography/nerve conduction study Bilateral tibial, fibular, median, and left ulnar CMAP distal latencies were severely prolonged, amplitudes were decreased and conduction velocities were slowed. There was evidence of temporal dispersion of the CMAPs in all nerves. The left median, ulnar, and radial SNAPs were absent. The left sural SNAP amplitude was decreased. This electrodiagnostic study showed evidence of a severe demyelinating sensorimotor polyneuropathy with axon loss. CMAP: compound motor action potential; SNAP: sensory nerve action potential

The patient presented for EMG/NCS and was noted on physical examination to display evidence of weakness of the bilateral deltoids (4/5), wrist flexion/extension (4/5), finger flexion/extension (4-/5), and foot dorsiflexion (right 4/5, left 5-/5). Additionally, there were absent reflexes except for 1+ at bilateral patella and sensory examination was notable for decrease of pinprick and vibration sensation in the feet. The EMG/NCS showed evidence of a severe demyelinating sensorimotor polyneuropathy with axon loss, with temporal dispersion noted (Table 1, Figure 1). The needle examination showed evidence of abnormal spontaneous activity in the right first dorsal interosseous, flexor carpi radialis, and abductor pollicis brevis muscles with chronic reinnervation changes in the right extensor digitorum, first dorsal interosseous, abductor pollicis brevis, and tibialis anterior muscles. Based on this, additional workup was performed, including laboratory testing and cerebrospinal fluid analysis (Table 2). She was treated with intravenous immune globulins (IVIG) 400 mg/kg/day for five days followed by maintenance with 1g/kg divided over three days every three weeks. She also underwent physical and occupational therapy.

**Table 1:** Initial nerve conduction study values NCS: nerve conduction study

Motor NCS	Latency (ms)	Amplitude (mV)	Velocity (m/s)
Left Median	22.6 (ref <4.2)	0.5 (ref >3.5)	-
Right Median	19.2 (ref <4.2)	0.5 (ref >3.5)	-
Left Ulnar	23.2 (ref <3.4)	1.1 (ref >6)	18 (ref >49)
Right Peroneal	8 (ref < 5.5)	1.5 (ref >2.5)	12 (ref >40)
Left Peroneal	8 (ref < 5.5)	1.5 (ref >2.5)	29 (ref >40)
Right Tibial	8.3 (ref <6)	1 (ref >2.9)	-
Left Tibial	23.4 (ref <6)	1 (ref >2.9)	-
Sensory NCS Anti	Onset Latency (ms)	Base-Peak Amplitude (uV)	Velocity (m/s)
Left Ulnar	No response	No response	No response
Left Median	No response	No response	No response
Left Radial	No response	No response	No response
Left Sural	3 (ref <4.4)	3.7 (ref >5)	43 (ref>38)

**Table 2:** Laboratory workup

Testing	Patient Values	Reference Range
C-Reactive Protein	<0.3	0.0 - 1.0 mg/dL
Zinc	60	60 - 130 mcg/dL
Total Protein, Serum	7.2	6.0 - 8.3 g/dL
Alpha-1-Globulin	0.30	0.10 - 0.30 g/dL
Alpha-2-Globulin	0.70	0.60 - 1.00 g/dL
Gamma Globulin	1.10	0.70 - 1.50 g/dL
Albumin Electrophoresis	4.30	3.50 - 4.70 g/dL
Beta Globulin	0.80	0.70 - 1.20 g/dL
Serum protein electrophoresis (SPEP) Interpretation	Normal Electrophoretic Pattern	
Platelets	291	140 - 446 x 1000/µL
Sedimentation Rate (ESR)	11	0 - 20 mm/hr
Prothrombin Time	10.3	9.4 - 12.0 seconds
International Normalized Ratio (INR)	0.97	0.88 - 1.14
Partial Thromboplastin Time (PTT)	25.5	23.0 - 31.0 seconds
Immunoglobulin A (IgA)	165	70 - 400 mg/dL
Immunoglobulin M (IgM)	78	40 - 230 mg/dL
Immunoglobulin G (IgG)	1,060	700 - 1,800 mg/dL
Immunofixation Electrophoresis Gel	No Distinct Band Detected	
Rheumatoid Factor	<10.0	0.0 - 14.0 IU/mL
Myeloperoxidase Antibody	<0.2	0.0-0.9 AI
Proteinase 3 Immunoglobulin G Antibody	<0.2	0.0-0.9 AI
Voltage-Gated Potassium Channel Antibody	<80	<80 pmol/L
Acetylcholine Receptor Antibody	<0.30	
Striated Muscle Antibody	Negative	Negative
Lyme Antibody	0.08	0.00-0.89 Index
Glioblastoma Multiforme Antibody	<0.2	0.0-0.9 AI
Cerebrospinal fluid (CSF)		
Appearance, CSF 1	Clear	Clear
Color, CSF 1	No Xanthochromia	No Xanthochromia
Red Cells/uL, CSF #1	570	
Nucleated Cells/uL, CSF #1	1	
CSF	Tube 4	
Appearance, CSF 4	Clear	Clear
Color, CSF 4	No Xanthochromia	No Xanthochromia
Red Cells/uL, CSF #4	191 (H)	None Cells/uL
Nucleated Cells/uL, CSF #4	1	0 - 5 Cells/uL
Glucose, CSF	46	40 - 75 mg/dL
Protein, Total CSF	39.0	15.0 - 45.0 mg/dL
Treponema Pallidum Antibody Total, Serum	<0.2	0.0 - 0.8 AI
Lyme Antibody, CSF	<0.01	Index
Antinuclear Antibody 1 (Hu), Indirect Immunofluorescence Assay	Negative	Negative
Antinuclear Antibody 2 (Ri), Indirect Immunofluorescence Assay	Negative	Negative
Antinuclear Antibody 3, Indirect Immunofluorescence Assay	Negative	Negative
Purkinje Cell Cytoplasmic Antibody Type 1 (Yo), Indirect Immunofluorescence Assay	Negative	Negative
Amphiphysin Antibody, Indirect Immunofluorescence Assay	Negative	Negative
Collapsin Response Mediator Protein 5 (CRMP 5)/CV2 Antibody, Indirect Immunofluorescence Assay	Negative	Negative
Voltage-Gated Calcium Channel Type N Antibody	<54	<54 pmol/L
Alpha 3 Ganglionic Acetylcholine Receptor Antibody	<53	<53 pmol/L
Anti-Glial Nuclear Antibody (AGNA)/SOX1 Antibody, Indirect Immunofluorescence Assay, CSF	Negative	Negative
Amphiphysin Antibody, Indirect Immunofluorescence Assay, CSF	Negative	Negative
Angiotensin-Converting Enzyme, CSF	7	<=15 U/L
Antinuclear Antibody 1 (Hu), Indirect Immunofluorescence Assay, CSF	Negative	Negative
Antinuclear Antibody 3, Indirect Immunofluorescence Assay, CSF	Negative	Negative
Aquaporin-4 Antibody, Indirect Immunofluorescence Assay, CSF	Negative	Negative
Copper	106	70 - 175 mcg/dL
Collapsin Response Mediator Protein 5 (CRMP 5)/CV2 Antibody, Indirect Immunofluorescence Assay, CSF	Negative	Negative
Glutamic Acid Decarboxylase Antibody, Indirect Immunofluorescence Assay, CSF	Negative	Negative
Ganglioside Asialo-GM 1 Antibody, Immunoglobulin G, Enzyme-Linked Immunosorbent Assay	<1:100	<1:100 titer
Ganglioside Asialo-GM 1 Antibody, Immunoglobulin M, Enzyme-Linked Immunosorbent Assay	<1:1600	< Or = 1:1600 titer
Ganglioside GD1A Antibody, Immunoglobulin G, Enzyme-Linked Immunosorbent Assay	<1:100	<1:100 titer
Ganglioside GD1B Antibody, Immunoglobulin G, Enzyme-Linked Immunosorbent Assay	<1:100	<1:100 titer
Voltage-Gated Calcium Channel Antibody	<30	<30 pmol/L

**Figure 2:**
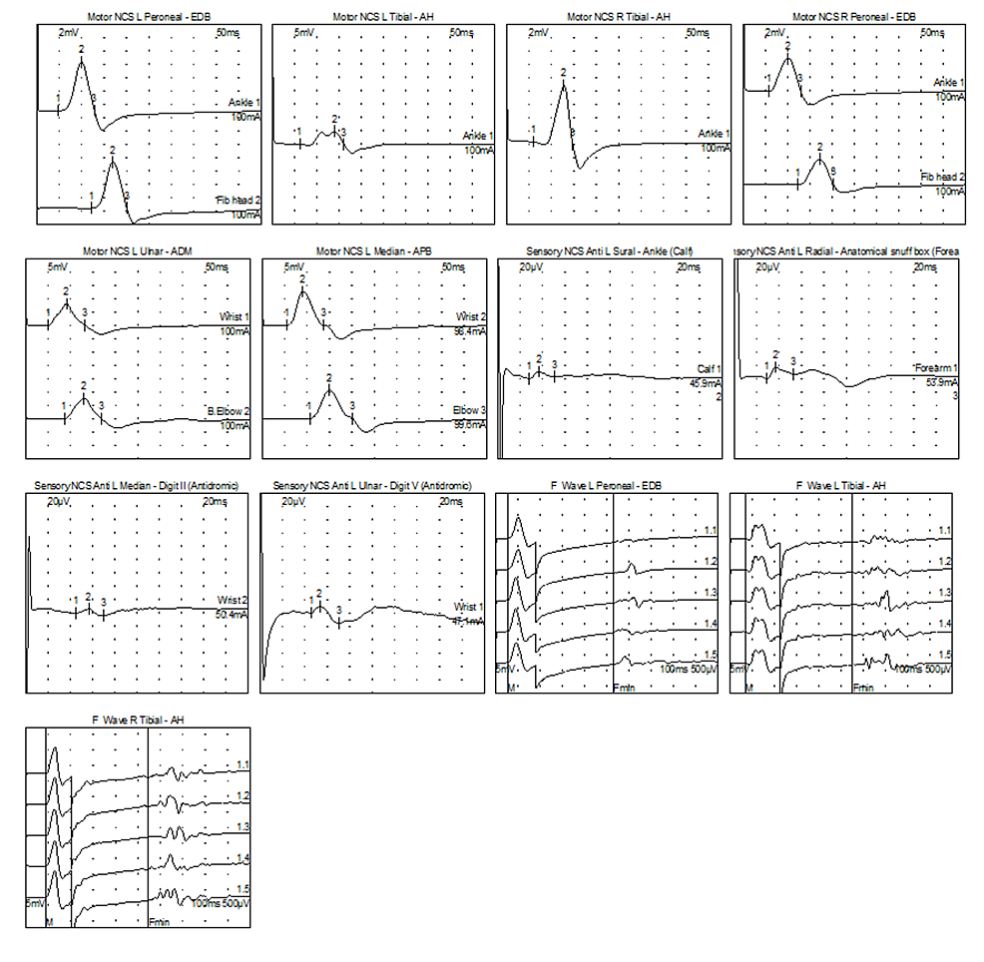
Follow-up EMG/NCS The left median CMAP distal latency was prolonged and the conduction velocity was slowed. The left ulnar, left tibial and right fibular CMAPs distal latencies were prolonged. The left ulnar, median and radial SNAP distal latencies were prolonged, amplitudes were decreased and the conduction velocities were slowed. The left sural SNAP was normal. This electrodiagnostic study showed evidence of a diffuse demyelinating sensorimotor polyneuropathy. CMAP: compound motor action potential; SNAP: sensory nerve action potential

At the follow-up visit two months later, she had improved significantly and was able to perform all of her activities of daily living following IVIG therapy. She had no adverse reactions to the therapy. Neurologic examination was notable for full strength in both upper and lower extremities as well as 2+ reflexes throughout. The plan was to continue IVIG and repeat an EMG/NCS to assess for any electrodiagnostic changes. Follow-up EMG/NCS showed evidence of a diffuse demyelinating sensorimotor polyneuropathy, though when compared to the prior study there was a significant improvement in the degree of demyelination and axon loss (Figure 2).

## Discussion

This case presentation highlights the difficulty in diagnosing CIDP as well as the importance of close clinical follow-up and physical examination. CIDP can be a difficult diagnosis and prompt treatment can result in favorable outcomes and limited morbidity, as shown in this patient. With virtual visits becoming a standard part of clinical care for the foreseeable future, it is important to systematically and comprehensively evaluate patient symptoms. In this case, the patient presented with symptoms of neuropathy in the distal extreme ities which had progressed in the months prior to presentation. A detailed history coupled with the limited virtual examination allowed for the correct workup to be ordered including laboratory testing and electrodiagnostic studies. The video modality prevented the detection of subtle signs of distal sensorimotor polyneuropathy like vibratory sensation loss, mild proprioceptive deficits, absence of reflexes, distal muscle weakness without atrophy (sign of conduction block). The time period between the virtual visit and in person examination was limited, allowing for expedited assessment of the patient’s physical symptoms by a neuromuscular physician. However, establishing the precise diagnosis was difficult in the absence of a face-to-face physical examination. This made the case appear less urgent, which could have resulted in a poor outcome.

Electrodiagnostic findings of CIDP can include slowing of conduction velocities, prolongation of distal motor latencies, temporal dispersion, conduction block, and prolongation of F-waves [5]. Temporal dispersion is a reduction in proximal compound motor action potential (CMAP) amplitude compared with distal CMAP amplitude when the proximal CMAP duration increases by > 20% This patient’s initial EMG/NCS shows classic findings of demyelination with prominent evidence of temporal dispersion in all motor nerves.

The electrodiagnostic findings coupled with the physical examination were consistent with a diagnosis of CIDP, but it is important to rule out any other causes of muscle weakness [5]. A thorough workup includes CSF analysis coupled with comprehensive laboratory testing, as was done in this patient (Table 2).

The intravenous immunoglobulin (IVIG) for the treatment of chronic inflammatory demyelinating polyradiculoneuropathy (ICE study) demonstrated the safety and efficacy of IVIG in CIDP patients and, as seen in this patient, can result in improved clinical outcomes [6]. While there can be significant clinical improvement, electrodiagnostic findings can improve at a slower rate, again demonstrated by this case.

When evaluating patients with neuropathy or other neurologic conditions where examination is crucial, it becomes important to assess motor function in any manner possible. Additionally, a comprehensive history can result in clues about motor deficits including asking patients about difficulties lifting objects, if they are able to stand on their toes, difficulties opening jars or buttons, and difficulties with fine motor tasks. A thorough history is not a substitute for an exhaustive and nuanced neurologic examination but can serve to provide important clinical information. Providers should aim to keep a broad differential diagnosis and order testing in a stepwise yet complete manner. Lastly, attempts should be made to limit time to follow-up to ensure close clinical monitoring.

## Conclusions

Diagnosing CIDP can be challenging for providers and the use of virtual visits can add to the complexity. It is crucial to have a broad differential diagnosis in patients who cannot be physically examined and ensure close follow-up to monitor for symptom change. Virtual visits have led to improvements in patient access to healthcare and play an important role in the current practice of healthcare, but care should be given to continue to provide excellent clinical care. This case highlights the limits of tele-neurology and describes the workup and testing required for a diagnosis of CIDP as well as strategies for workup when utilizing virtual visits.
